# Removal rates and energy demand of the electrochemical oxidation of ammonia and organic substances in real stored urine

**DOI:** 10.1039/c7ew00014f

**Published:** 2017-03-10

**Authors:** Hanspeter Zöllig, Annette Remmele, Eberhard Morgenroth, Kai M. Udert

**Affiliations:** a Eawag, Swiss Federal Institute of Aquatic Science and Technology, 8600 Dübendorf, Switzerland. E-mail: kai.udert@eawag.ch; Fax: +41 58 765 5808; Tel: +41 58 765 5360; b ETH Zürich, Institute of Environmental Engineering, 8093 Zürich, Switzerland

## Abstract

The separate collection and treatment of urine allows for an environmentally friendly and cost-efficient management of the nutrients contained in urine. The primary goal should be to recover all these nutrients. However, in some cases it will be economically or ecologically more sensitive to recover only the phosphorus, while nitrogen is removed together with organic substances (measured as chemical oxygen demand, COD) and pathogens. In this study, we investigated the use of galvanostatic electrolysis for the removal of nitrogen and COD from real stored urine. Non-active type boron-doped diamond (BDD) and active type thermally decomposed iridium oxide film (TDIROF) anodes were evaluated using batch experiments. On both anodes, ammonia was exclusively removed by indirect oxidation with active chlorine (AC:Cl_2_, HClO, and ClO^−^). As a consequence, ammonia was not completely removed, if chlorine was consumed by competing processes. While COD was present, ammonia removal was faster on TDIROF (227 ± 16 gN m^−2^ d^−1^ at 20 mA cm^−2^) than on BDD (43 ± 20 gN m^−2^ d^−1^ at 20 mA cm^−2^). The reason for the slower ammonia removal on BDD was the enhanced reaction of AC with organic molecules. In fact, hydroxyl radicals broke organic molecules down to shorter chain molecules which reacted with most of the AC leaving only little AC for the oxidation of ammonia. This preferential oxidation of organics resulted in very high COD removal rates on BDD (above 420 gCOD m^−2^ d^−1^ at 20 mA cm^−2^ for COD concentrations above 1000 mgCOD L^−1^). A main drawback of electrolysis with both anodes was the high energy demand (BDD: 55 W h gCOD^−1^ and 766 W h gN^−1^ for 90% and 6% removal, respectively. TDIROF: 67 W h gCOD^−1^ and 77 W h gN^−1^ for 30% and 40% removal. All at 20 mA cm^−2^). It can be concluded that BDD and TDIROF anodes could be combined in series for a fast, complete, and more energy efficient electrochemical urine treatment: COD could be removed on BDD before the residual ammonia would be removed on TDIROF.

Water impact

The separate collection and treatment of urine is a promising approach for innovative sanitation systems. For on-site urine treatment electrolysis could be a robust and compact technology. The article shows that electrolysis can simultaneously remove nitrogen and organic substances at high rates from real stored urine. However, this comes with a price: high energy needs and harmful byproducts.

## 1 Introduction

Urine is a concentrated source of nutrients and can be collected separately from the other wastewater streams in waterless urinals or urine-diverting toilets. When fresh urine is excreted by humans, most of the nitrogen (85%) is present as urea and the pH is about 6.4 ± 1.1.^1^ In stench traps, pipes, and storage tanks, bacteria producing the enzyme urease hydrolyze urea quickly (ureolysis) to bicarbonate and ammonia.^2^ The result is so called ureolyzed or stored urine with a pH around 9. In stored urine, 90% of the nitrogen is present in the form of ammonia (NH_4_^+^ and NH_3_). Urine treatment processes mostly have to treat stored urine since urine is hardly ever collected under sterile conditions.

Several processes were proposed for nutrient recovery from stored urine.^3^ Some processes, such as evaporation^4^ or reverse osmosis,^5^ enable an almost complete separation of all nutrients and water whereas others, such as electrodialysis^6^ or freeze and thaw methods,^7^ leave considerable concentrations of the nutrients in the treated effluent (>300 mgN L^−1^, >60 mgP L^−1^, >800 mgCOD L^−1^). Very high nitrogen and COD concentrations, actually close to the ones in the raw urine, remain in the supernatant of the struvite process.^8^ These residual compounds need to be eliminated from the supernatant of a urine treatment and pathogens must be inactivated.

Electrolysis could be a suitable technology for these purposes especially if compact reactors are desirable for on-site treatment.^9^ Organic substances as well as urea were successfully removed from synthetic fresh urine for environmental protection.^10,11^ Other studies used fresh urine as a source of urea for hydrogen production.^12,13^ It was also shown that electrolysis kills pathogens in fresh urine due to the produced AC (Cl_2_, HClO, ClO^−^).^14^ Additional advantages are that the technology is stand alone, requires low maintenance, and does not rely on the addition of chemicals.^9^ Furthermore, electrolysis is easy to automate and the treatment progress can be monitored online because the current gives direct information of the reaction rates.^15^

Only few publications evaluate electrolysis for the removal of ammonia and COD from stored urine. Zheng *et al.* reported high ammonia oxidation rates on graphite anodes applying high constant currents (50 mA cm^−2^).^16^ This very likely was through indirect ammonia oxidation by AC because it was shown recently that direct ammonia oxidation results in much slower removal rates.^17^ Furthermore, the very high current density must have resulted in the corrosion of the graphite anode^18^ and the production of chlorination byproducts.^19^ Zheng *et al.* reported successful indirect ammonia oxidation on a dimensionally stable anode (DSA, Ti/RuO_2_–IrO_2_–TiO_2_) until specific charges of 3.6 A h L^–1^.^20^ However, Amstutz *et al*. observed the complete inhibition of indirect ammonia oxidation on a similar DSA (Ti/IrO_2_) when higher specific charges were applied (10 to 80 A h L^−1^) due to competing carbonate oxidation.^21^ In the latter two studies, no COD removal was reported but it is known from many other studies in different kinds of wastewater that AC plays an important role for COD removal if chloride is present.^22^ It remains unclear if complete, simultaneous ammonia and COD removal by electrolytic oxidation is feasible in stored urine and what would be the best operating conditions.

In this work, we discuss three important aspects of simultaneous electrochemical removal of COD and ammonia from real stored urine. In galvanostatic electrolysis experiments in batch reactors we assessed the removal rates of COD and ammonia, determined the necessary specific energy demand, and evaluated the composition of the treated urine. Furthermore, the impacts of three important operating parameters were evaluated: the electrode material (BDD and TDIROF), the current density, and the composition of the raw urine by working with low and high-concentration urine from two different sources. Thus, the presented work adds important knowledge to judge the practical applicability of electrolysis to simultaneously remove nitrogen and organic substances from stored urine.

## 2 Materials and methods

### 2.1 Urine electrolysis

The details to the setup of the electrochemical cell were described in a previous publication.^19^ The most important settings are given here for convenience.

An undivided glass cell (400 mL) was equipped either with a boron-doped diamond (Si/BDD, Adamant Technologies SA, La Chaux-de-Fonds, Switzerland) or a thermally decomposed iridium oxide film (TDIROF) ^23^ anode with 20 cm^2^ of exposed surface area. The cathode with an equivalent surface area was made of steel (X5CrNi18-10, Hans Kohler AG, Zürich, Switzerland). The distance between the electrodes was between 9 and 10 mm. A mercury/mercurous sulfate (MSE) reference electrode was employed to measure the anode potential (*E*_A_ in V) and was placed in a glass-blown Luggin capillary filled with saturated K_2_SO_4_. Temperature and pH were measured continuously (SenTix 41 connected to pH 196, WTW, Weilheim, Germany) and recorded with a data logger. Conductivity was measured with a handheld meter (TetraCon 325 connected to Cond 340i, WTW, Weilheim, Germany).

The electrolysis cell was filled with 350 mL of stored urine which was unequally diluted with intruding flushing water during collection. Only slightly diluted men's urine (because of urinals) and more strongly diluted women's urine were used from the collection tanks at Eawag (Table 1). Electrolysis was performed with a potentiostat (PGU 10V-1A-IMP-S, Ingenieurbüro Peter Schrems, Münster, Germany) which registered *E*_A_ under galvanostatic control at *j* = 10, 15 and 20 mA cm^−2^. A magnetic stirrer ensured turbulence in the reactor and the temperature was controlled with a thermostat (K3 DS, Colora Messtechnik GmbH, Lorch, Germany) at a fixed temperature indicated for each experiment.

**Table 1:** Composition of ureolyzed urine from the women's urine storage tank (low-concentration urine) and the men's urine storage tank (high-concentration urine) at Eawag. The number of measurements was usually six

		Low-concentration urine		High-concentration urine	
		Average	Std. dev.	Average	Std. dev.
Total COD	(mg L^−1^)	1710	50	4510	290
Total ammonia N	(mg L^−1^)	1860	40	2790	170
Nitrite N	(mg L^−1^)	<0.15	—	<10	—
Nitrate N	(mg L^−1^)	<1	—	<10	—
Chloride	(mg L^−1^)	1250	130	3800	150
Total phosphate P	(mg L^−1^)	88	9	242	8
Sulfate	(mg L^−1^)	234	25	822	45
Total inorganic carbon	(mg L^−1^)	903*^a^*	—	—	—
Conductivity	(mS cm^−1^)	15.1*^a^*	—	30.1	1.1
pH	(—)	9.1*^b^*	0.1	9	0.1
COD/ammonia-N	(mg mg^−1^)	0.92	0.04	1.62	0.20
COD/Cl^−^	(mg mg^−1^)	1.38	0.13	1.18	0.05
Ammonia-N/Cl^−^	(mg mg^−1^)	1.50	0.15	0.74	0.05

a One measurement.

b Five measurements.

### 2.2 Chemical analysis

Aliquots (∼13 mL) were taken with a syringe through a needle permanently installed in the glass lid of the reactor. After the total COD was analyzed, the samples were filtered with glassfiber filters (0.45 μm, Chromafil GF/PET, Macherey-Nagel, Düren, Germany). Chloride, phosphate, sulfate, nitrite, and nitrate were analyzed by ion chromatography (881 compact IC pro, Metrohm, Herisau, Switzerland). Ammonia and total COD were measured photometrically with cuvette tests (LCK 303 and LCK 314/614, Hach Lange, Berlin, Germany). The total inorganic carbon was measured once with a total inorganic/total organic carbon analyzer (TOC-L, Shimadzu, Kyoto, Japan) according to manufacturers' protocol in a sample directly taken from the women's storage tank. The standard deviations of the wet chemical analyses were less than 5%.

### 2.3 Calculations

#### 2.3.1 Removal and production rates

In galvanostatic electrolysis, first order kinetics are typical for mass transport limited reactions and zero order kinetics in case of electron transfer limited reactions.^24^ Therefore, the data was evaluated differently depending on the dominating mass transfer regime.

In this study, strongly mass transfer controlled conditions were only observed for COD degradation on BDD for which the area specific removal rate *r*_COD_ (g m^−2^ d^−1^) can merely be expressed at a certain COD concentration in the bulk (*S*_COD,∞_ (mg L^−1^)):^24^

$$r_{COD}=\frac{dS_{COD,\infty }\left(t\right)}{dt}\cdot \frac{V}{A}=-k_{m,COD}\cdot S_{COD,\infty }\left(t\right)$$ (1)

Herein, *A* (m^2^) is the electrode surface area, *V* (m^3^) is the volume of the electrolyte, *t* is the time and *k*_m,COD_ (m s^−1^) is the apparent mass transfer coefficient for COD.

After rearrangement and integration eqn (1) becomes:

$$\ln\left(S_{COD,\infty }\left(t\right)\right)=-k_{m,COD}\cdot \frac{A}{V}\cdot t+\ln\left(S_{COD,\infty }\left(0\right)\right)=-p\cdot t+\ln\left(S_{COD,\infty }\left(0\right)\right)$$ (2)

Thus, *k*_m,COD_ was estimated with the initial electrolyte volume *V*_init_ (m^3^) according to eqn (3):

$$k_{m,COD}=\frac{p.V_{init}}{A}$$ (3)

The slope *p* was taken from the linear regression in the logarithmic plot of the COD concentration against *t* when COD removal apparently followed first order kinetics.

All other area specific removal or production rates *r*_x_ (g m^−2^ d^−1^) under electron transfer limitation were calculated from a linear regression of the mass (considering the sampling) *versus* time:

$$r_{x}=a_{x}\cdot \frac{1}{A}$$ (4)

where *a*_x_ (g d^−1^) is the slope.

#### 2.3.2 Current efficiencies and specific energies

Under the assumption that all ammonia, which was not converted to nitrate or nitrite, was oxidized to N_2_, the total current efficiency for ammonia oxidation (CE_NH_, in %) was estimated as:

$$CE_{NH}\left(t\right)=CE_{NO_{3}}{}^{-}\left(t\right)+CE_{NO_{2}}{}^{-}\left(t\right)+CE_{N_{2}}\left(t\right)$$ (5)

where

$$CE_{x}\left(t\right)=\frac{\Delta m_{x}\left(t\right)\cdot u_{e^{-},x}\cdot F}{M_{N}\cdot Q\left(t\right)}$$ (6)

and

$$2NH_{4}^{+}+3ClO^{-}\to N_{2}+3H_{2}O+3Cl^{-}+2H^{+}$$ (7)

Here, Δ*m*_x_(*t*) (gN) is the mass of substance x produced in the cell until time *t*, and υ_e_−_,x_ (mole^−^ molN^−1^) is the number of electrons used per mole of ammonia oxidized to nitrate (υ_e_−_,NO_3__− = 8) nitrite (υ_e_−_,NO_2__− = 6) or molecular nitrogen (υ_e_−_,N_2__ =3), respectively. *F* is the Faraday constant (96 485 C mole^−1^), *M*_N_ the molecular weight of nitrogen (14 gN molN^−1^) and *Q*(*t*) (C) the transported charge, calculated from the current *I* (A) as:

$$NH_{4}{}^{+}+4ClO^{-}\to H_{2}O+NO_{3}{}^{-}+4Cl^{-}+2H^{+}$$ (8)

The current efficiency for organic substance removal (CE_COD_) was estimated based on COD measurements:

$$NO_{3}{}^{-}+H_{2}O+2e^{-}↔NO_{2}{}^{-}+2OH^{-}$$ (9)

where Δ*m*_COD_(*t*) (gO_2_) is the mass of COD removed until a time *t*. TOD_e_− is the theoretical oxygen demand of one mole of electrons (8 gO2 mol^−1^).

The cumulative specific energy demand for the removal of total ammonia (x = NH) or COD (x = COD) *E*_sp,x_ (W h g^−1^) was calculated from the average electrical power $\bar{U\cdot I}$, which was calculated as the average of the product of the cell voltage *U* (V) times the current *I* (A) up to a time *t*:

$$NO_{2}{}^{-}+5H_{2}O+6e^{-}\to NH_{3}+7OH^{-}$$ (10)

where the factor 3600 converts seconds to hours.

## 3 Results

### 3.1 Electrolysis of high-concentration urine

#### 3.1.1 BDD

**Fig. 1:**
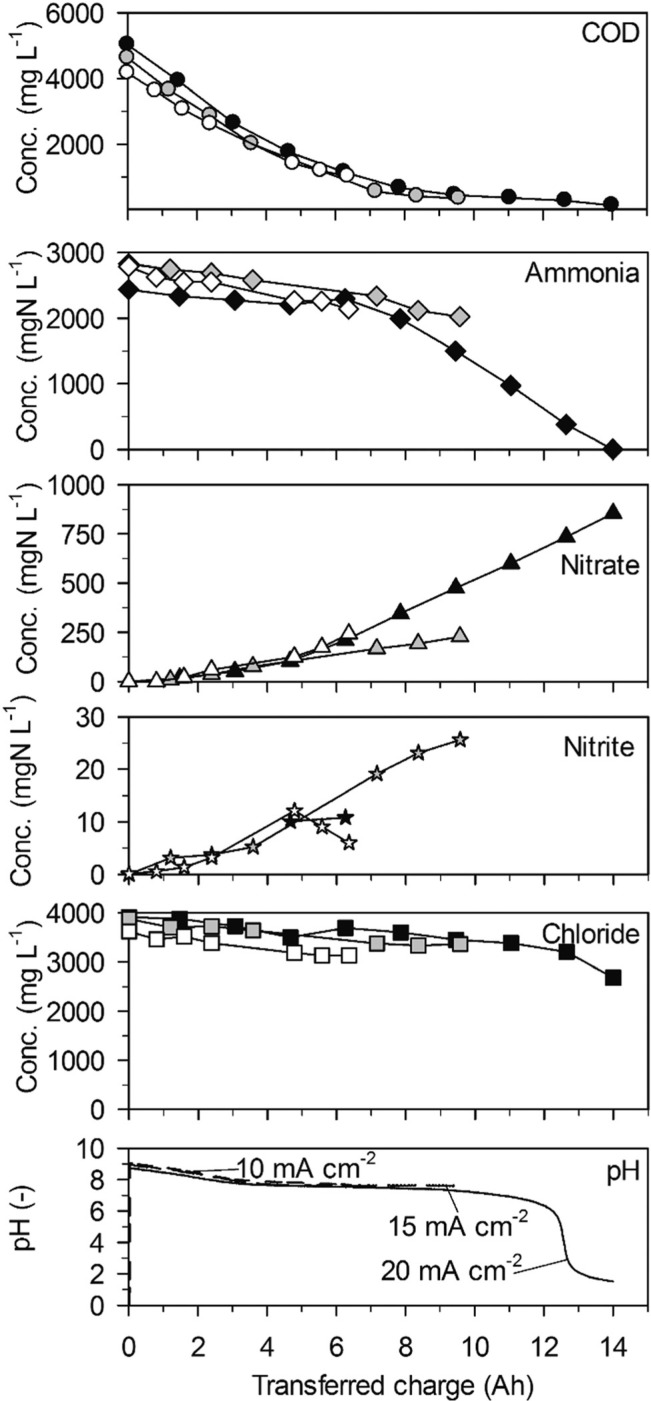
Galvanostatic electrolysis of high-concentration urine on BDD. Full symbols: 20 mA cm^−2^ (25.2 °C), grey symbols: 15 mA cm^−2^ (25.1 °C) and empty symbols 10 mA cm^−2^ (25.0 °C). Electrode gap: 9 mm.

The removal rates of COD were very high (Table 2, 421 ± 38 gCOD m^−2^ d^−1^, at 1000 mgCOD L^−1^ and 20 mA cm^−2^) but decreased with the transferred charge depending on the COD concentration (Fig. 1). This can be explained with a first order reaction of organic substances indicating mass transfer control of the process. The apparent mass transfer coefficients *k*_m,COD_ were in the same order of magnitude but showed a trend towards higher values with increasing current density indicating that the process was also electron transfer controlled.

**Table 2:** Rates of COD, chloride, and ammonia removal as well as the nitrate production rates in the initial linear ranges of the experiments. In case of the BDD anode, COD removal was not linear with transferred charge. Thus, the COD removal rates with BDD are given at 1000 mgCOD L^−1^ according to eqn (1) based on the calculated apparent mass transfer coefficients k_m,COD_. SD denotes the standard deviation

			Removal of organic substances				Chlorideremoval		Ammonia removal		Nitrate production	
			rCOD	SD	k_m,COD_	SD	*r*_Cl_−	SD	*r*_NH_	SD	*r*_NO_3_‐N_	SD
Urine type	Electrode	*j* (mA cm^−2^)	(g_COD_ m−^2^ d^−1^)		(m s^−1^)		(g_Cl_− m^−2^ d^−1^)		(g_N_ m^−2^ d^−1^)		(g_N_ m^−2^ d^−1^)	
High-concentration urine	BDD	20	421	38	4.9 × 10^–6^	4.4 × 10^−7^	94	14	43	20	51	9
		15	334	26	3.9 × 10^−6^	3.0 × 10^−7^	58	9	87	5	26	1
		10	190	4	2.2 × 10^−6^	4.9 × 10^−8^	59	6	71	6	28	2
	TDIROF	20	214	24			174	13	227	16	17	1
		15	180	24			84	10	184	7	17	1
		10	150	15			20	22	147	5	28	2
Low-concentration urine	BDD	20	693	38	8.0 × 10^−6^	4.4 × 10^–7^	268	9	—	—	95	2
		15	549	28	6.4 × 10^−6^	3.2 × 10^−7^	167	7	—	—	75	3
		10	467	26	5.4 × 10^−6^	3.0 × 10^−7^	65	5	—	—	40	2
	TDIROF	20	163	13			164	6	257	22	64	7
		15	133	2			185	22	183	27	55	6
		10	91	6			89	5	110	11	27	1

Ammonia was eliminated at much lower rates than COD in a first phase of the experiments up to a transferred charge of 6 A h (Table 2, 43 ± 20 gN m^−2^ d^−1^ at 20 mA cm^−2^, Fig. 1). The preferential oxidation of COD on BDD is also reflected by the high CE_COD_ (Fig. 3). At 20 mA cm^−2^ ammonia removal accelerated to a much higher rate (419 ± 17 gN m^−2^ d^−1^) when the COD was exhausted after a transferred charge of 6 A h. This resulted in complete ammonia removal up to a transferred charge of 14 A h. Simultaneously, chloride was removed at the same constant rate as in the first phase of the experiment (Fig. 1, Table 2). This corroborates that indirect oxidation of organic substances and ammonia were competing reactions on BDD with a preference for COD oxidation.

**Fig. 2:**
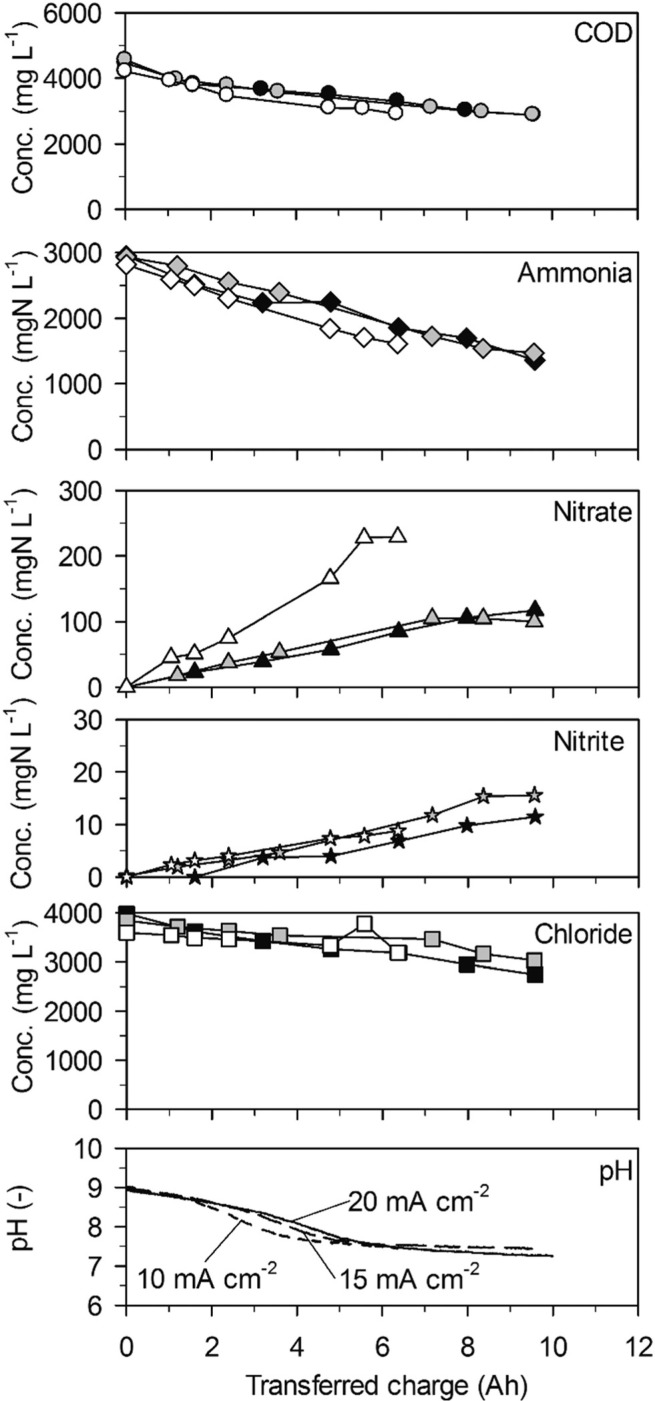
Galvanostatic electrolysis of high-concentration urine on TDIROF. Full symbols: 20 mA cm^−2^ (25.1 °C), grey symbols: 15 mA cm^−2^ (25.0 °C) and empty symbols 10 mA cm^−2^ (25.0 °C). Electrode gap: 10 mm.

The two phases of ammonia oxidation led to two different nitrate formation rates in the corresponding time periods at 20 mA cm^−2^. The nitrate formation rate was 51 ± 9 gN m^−2^ d^−1^ in the first phase until 6 A h. This rate increased to 105 ± 2 gN m^−2^ d^−1^ in the second phase. The final nitrate yields at current densities of 20, 15 and 10 mA cm^−2^ were 54.0 ± 37.5%, 26.7 ± 7.4% and 21.8 ± 13.12%, respectively. Simultaneously, only little nitrite accumulated (Fig. 1).

The pH value strongly decreased in the beginning of the experiments until a charge of about 4 A h was transferred (Fig. 1). From then on, the pH value remained at a level of 7.5 due to phosphate buffering (Table 1). At 20 mA cm^−2^, the buffer capacity of phosphate was exhausted after 12 A h of transferred charge which led to a pH drop to values below 2. This means that the consumption of alkalinity by anodic processes was stronger than alkalinity production by cathodic processes during the whole experiment.

#### 3.1.2 TDIROF

**Fig. 3:**
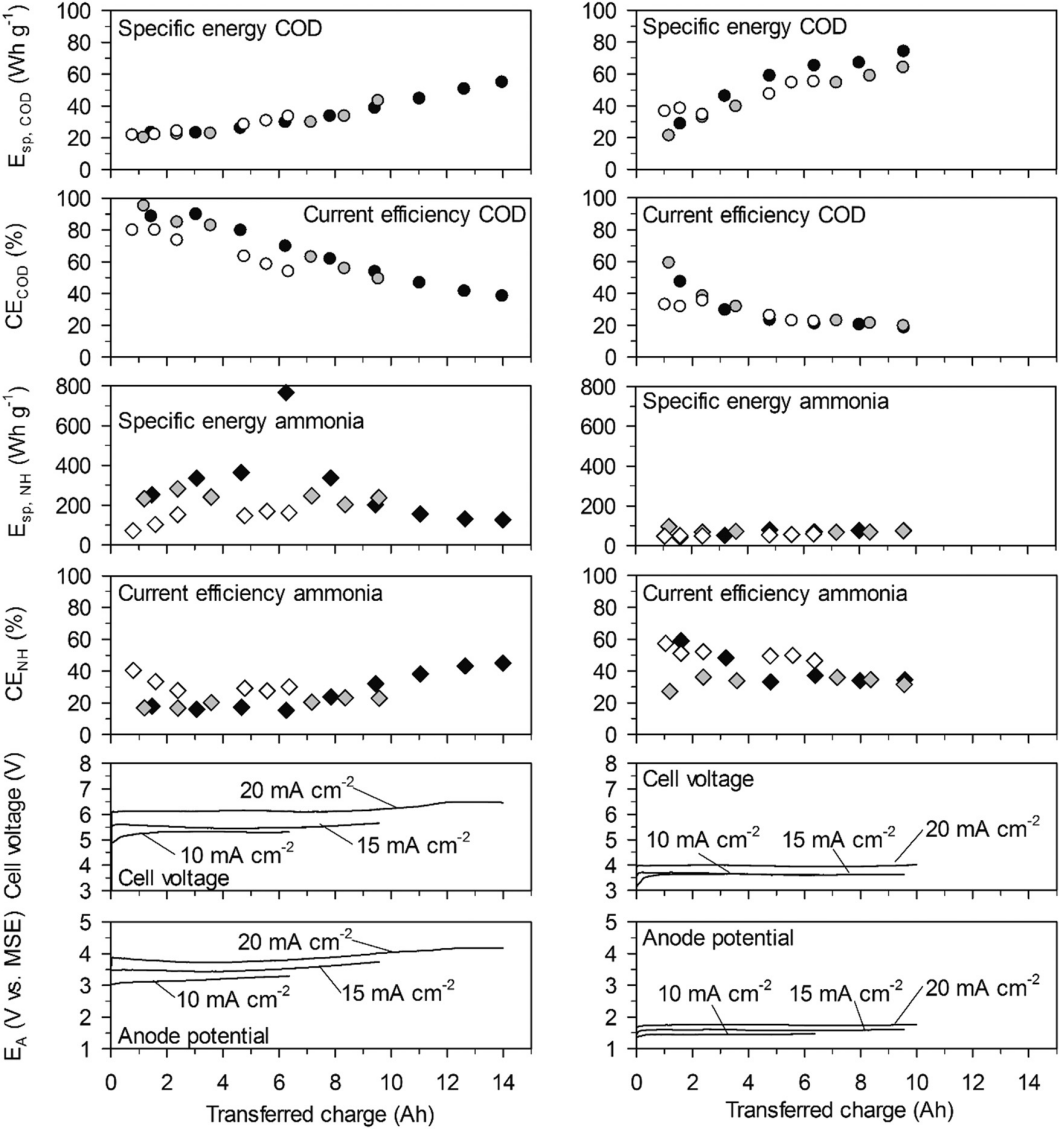
Energy consumption and current efficiency in high-concentration urine. Left: With the BDD anode. Right: With the TDIROF anode. Full symbols: 20 mA cm^−2^, grey symbols: 15 mA cm^−2^ and empty symbols 10 mA cm^−2^.

COD was removed at constant rates (Table 2, 214 ± 24 gCOD m^−2^ d^−1^ maximum at 20 mA cm^−2^) and did not depend on the bulk COD concentration indicating electron transfer control of COD removal on TDIROF (Fig. 2). Compared to the removal rates achieved on BDD, COD removal on TDIROF was much slower. However, the COD removal rates increased with current density which resulted from the CE_COD_ that did not depend on the current density (Fig. 3).

Also ammonia was removed at constant rates (Table 2, 227 ± 16 gN m^−2^ d^−1^ maximum at 20 mA cm^−2^) in parallel to the removal of COD (Fig. 2). The ammonia removal rates increased with the current density and were at least a factor two higher than on the BDD anode. This is in line with the higher chloride removal rates with increasing current density and also with the mostly higher chloride removal rates on TDIROF compared to BDD. At 20 mA cm^−2^, the chloride removal rate on TDIROF (174 ± 13 g m^−2^ d^−1^) was almost double the chloride removal rate observed on BDD (94 ± 14 gm^−2^ d^−1^) indicating the importance of indirect ammonia oxidation by AC.

Interestingly, the highest nitrate production rate of 28 ± 2 gN m^−2^ d^−1^ was observed at the lowest current density of 10 mA cm^−2^ (Table 2) and resulted in the highest nitrate yield of 18.0 ± 2.3% which is comparable to the yields observed in low-concentration urine (section 3.2.2). At 20 and 15 mA cm^−2^, the nitrate yields were comparatively low with 7.1 ± 1.2% and 9.5 ± 2.5%, respectively (Fig. 2). The reason for this could be stronger reduction of nitrate at higher current densities. Nitrite was only formed in small amounts leading to rather low concentrations (Fig. 2).

As with the BDD anode, the pH value dropped in the beginning of the experiments to reach a plateau at pH = 7.5 due to the phosphate buffer system (Fig. 2). About the same charge, 6 A h, was transferred to reach this level with current densities of 15 and 20 mA cm^−2^ whereas at 10 mA cm^−2^ only about 4 A h were needed. This corroborates the hypothesis that nitrate reduction was less prevalent at 10 mA cm^−2^: as we will discuss in section 4.1, cathodic nitrate reduction to nitrite and ammonia sets free hydroxyl ions and thereby buffers some of the protons released at the anode which slows down the decrease of pH.

#### 3.1.3 Current efficiency and specific energy consumption

On both electrodes *E*_sp,COD_ (cumulative value up to a time *t*) increased with the transferred charge (Fig. 3). This was mainly due to a decrease of CE_COD_ and not because of an increase in cell voltage.

On BDD at 20 mA cm^−2^, the initially high CECOD of more than 80% resulted in initial values for *E*_sp,COD_ as low as 23 W h gCOD^−1^ and for a COD elimination of 90% the *E*_sp,COD_ was only 55 W h gCOD^−1^. On TDIROF at 20 mA cm^−2^, the initial CECOD was below 60% and dropped quickly to values around 30%. As a consequence, the *E*_sp,COD_ was higher than on BDD and increased from 28 to 67 W h gCOD^−1^ to achieve a COD elimination of 30%. The higher *E*_sp,COD_ on TDIROF can be attributed solely to the low CE_COD_, as the cell voltage was clearly lower than with BDD. These results underline that COD removal is more efficient on BDD.

In contrast, the cumulative specific energy demand for ammonia removal, *E*_sp,NH_, was considerably lower on the TDIROF anode. At a current density of 20 mA cm^−2^ *E*_sp,NH_ increased from 42 to 77 W h gN^−1^ for the removal of about 40% of the ammonia. On BDD, the *E*_sp,NH_ was much higher and culminated in a maximum value of 766 W h gN^−1^ at 20 mA cm^−2^, after 6.25 A h were transferred and only 6% of the ammonia was removed. The reason for these high *E*_sp,NH_ values on BDD was the low CE_NH_. This observation underlines the better degradability of ammonia on TDIROF.

### 3.2 Electrolysis of low-concentration urine

#### 3.2.1 BDD

**Fig. 4:**
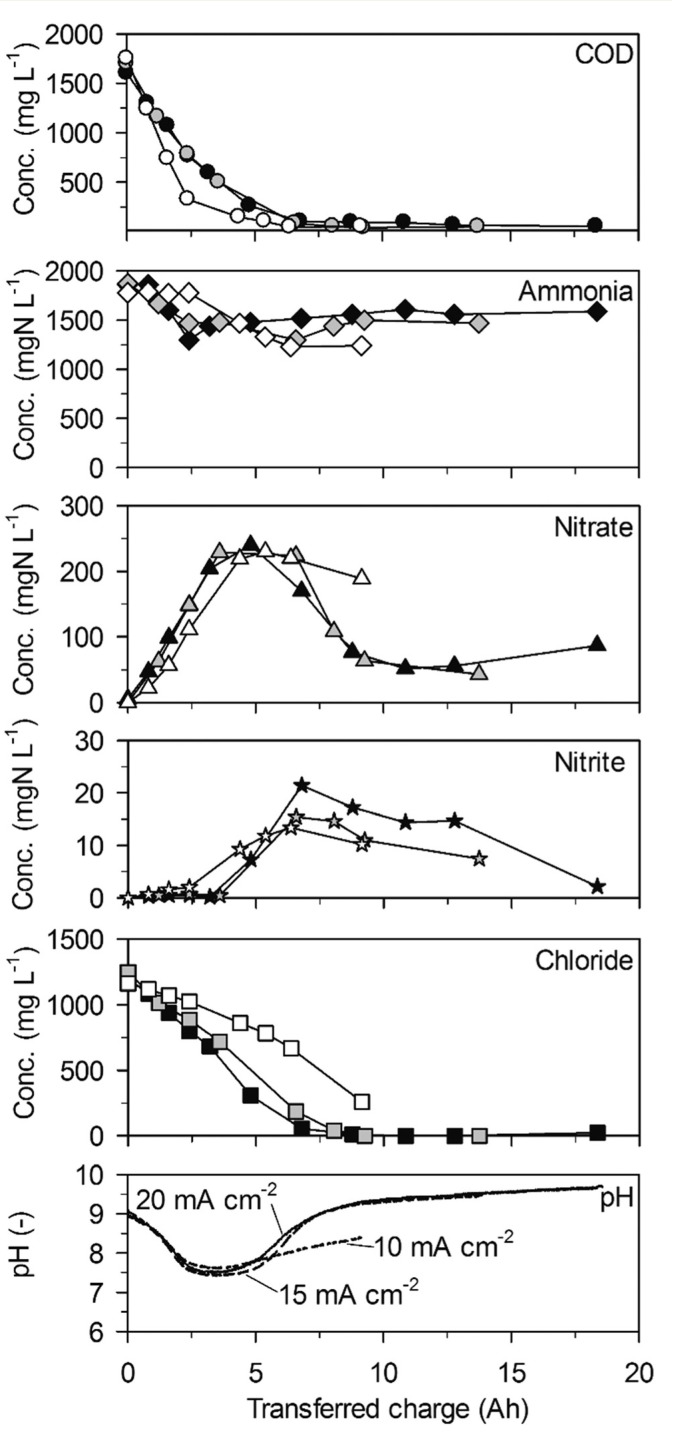
Galvanostatic electrolysis of low-concentration urine on BDD. Full symbols: 20 mA cm^−2^ (16.5 °C), grey symbols: 15 mA cm^−2^ (16.6 °C) and empty symbols 10 mA cm^−2^ (16.2 °C). Electrode gap: 9 mm.

In analogy to high-concentration urine, the COD removal was exponential as a function of the transferred charge but led to an almost complete COD removal (Fig. 4).Only about 50 mgCOD L^−1^ of recalcitrant COD remained at all current densities. The apparent *k*_m,COD_ values were increasing with the current density and were at a higher level than what was observed in high-concentration urine at the corresponding current density (Table 2). The faster COD removal was probably caused by higher AC concentrations which were the result of faster chloride oxidation (Table 2).

Ammonia was not removed when no chloride was present anymore towards the end of the experiments (Fig. 4, 15 and 20 mA cm^−2^). However, the constant nitrate formation (Table 2, 95 ± 2 gN m^−2^ d^−1^ at 20 mA cm^−2^) in the beginning of the experiments indicated ammonia oxidation to nitrate when chloride was present. Thus, it can be concluded that ammonia was only oxidized by AC. Unfortunately, it was not possible to calculate ammonia removal rates because the few ammonia data points in the useful range at the beginning of the experiments did not always show a clear trend.

The decreasing nitrate concentrations between 5 and 9 A h clearly indicated that nitrate was not only produced from ammonia but was also cathodically reduced. Nitrate reduction to ammonia can explain the increase of ammonia after 5 A h. The concentrations of nitrite were in a similar range to those in high-concentration urine but showed an intermediate peak at about 7 A h.

Again, the pH value declined from 9 to 7.5 within the first 2.5 A h of transferred charge. This was faster than in highconcentration urine and correlated with a stronger net nitrate production (Table 2). After 5 A h, when net nitrate reduction to ammonia set in, the pH value increased strongly at current densities of 15 and 20 mA cm^−2^. At 10 mA cm^−2^, however, the pH value increased more slowly in accordance with a slower net nitrate removal. This effect proves the importance of the interplay of anodic oxidation and cathodic reduction processes of nitrogen species for the course of pH.

#### 3.2.2 TDIROF

**Fig. 5:**
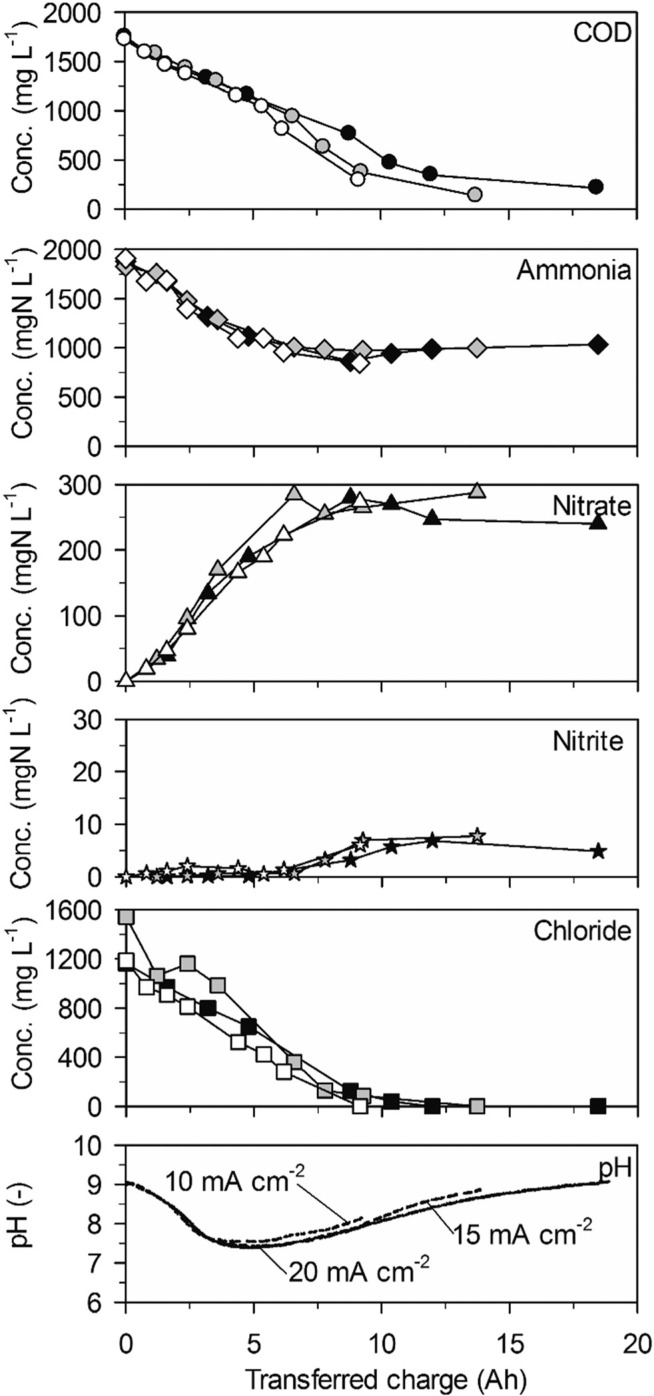
Galvanostatic electrolysis of low-concentration urine on TDIROF. Full symbols: 20 mA cm^−2^ (16.4 °C), grey symbols: 15 mA cm^−2^ (16.4 °C) and empty symbols 10 mA cm^−2^ (16.6 °C). Electrode gap: 10 mm.

The removal of COD occurred at constant but slightly lower rates than in high-concentration urine as long as the COD concentration was higher than about 800 mgCOD L^−1^ (Fig. 5). At lower COD concentrations, the COD removal rates decreased steadily. This transition of the kinetic regime coincided with the chloride being exhausted which indicated that indirect oxidation with AC played an important role during COD removal at constant rates. When chloride was used up, direct oxidation of organic substances was probably responsible for the ongoing COD removal.^22,25^

Ammonia was oxidized steadily to nitrate at comparable rates as in high-concentration urine (Table 2, 257 ± 22 gN m^−2^ d^−1^ maximum at 20 mA cm^−2^) as long as chloride concentrations were higher than approximately 500 mg L^−1^ (Fig. 5). However, ammonia removal slowed down considerably when chloride concentrations dropped to lower values. After a transition phase, ammonia removal stopped completely when no chloride was present anymore. Thus, a lack of chloride resulted in incomplete ammonia removal on BDD and TDIROF (Fig. 5).

The phases of net nitrate production (Table 2, 64 ± 7 gN m^−2^ d^−1^ maximum at 20 mA cm^−2^) were in accordance with the periods of ammonia oxidation (Fig. 5). Thereby, the higher production rates than in high-concentration urine led to higher nitrate yields of 25.9 ± 3.1%, 34.0 ± 6.4% and 19.8 ± 5.5% at 20, 15 and 10 mA cm^−2^, respectively. Similar to the experiments on BDD in low-concentration urine, a slight nitrate removal and a simultaneous increase of the ammonia concentration were observable at 20 mA cm^−2^ when no chloride was present anymore. This, again, indicates nitrate reduction. Nitrite only appeared when chloride concentrations dropped below approximately 500 mg L^−1^ and did not exceed 8 mgN L^−1^.

Similar to the previous experiments, a pH drop was observed during dominant ammonia oxidation to nitrate (Fig. 5). The same minimum pH value of 7.5 was reached but after more transferred charge than with the BDD anode in low-concentration urine. This was in accordance with the slower nitrate production rates on TDIROF (Table 2). In the following, the slowly increasing pH value may be attributed to a slower reduction of nitrate to ammonia compared to the observation on the BDD anode.

#### 3.2.3 Current efficiency and specific energy consumption

**Fig. 6:**
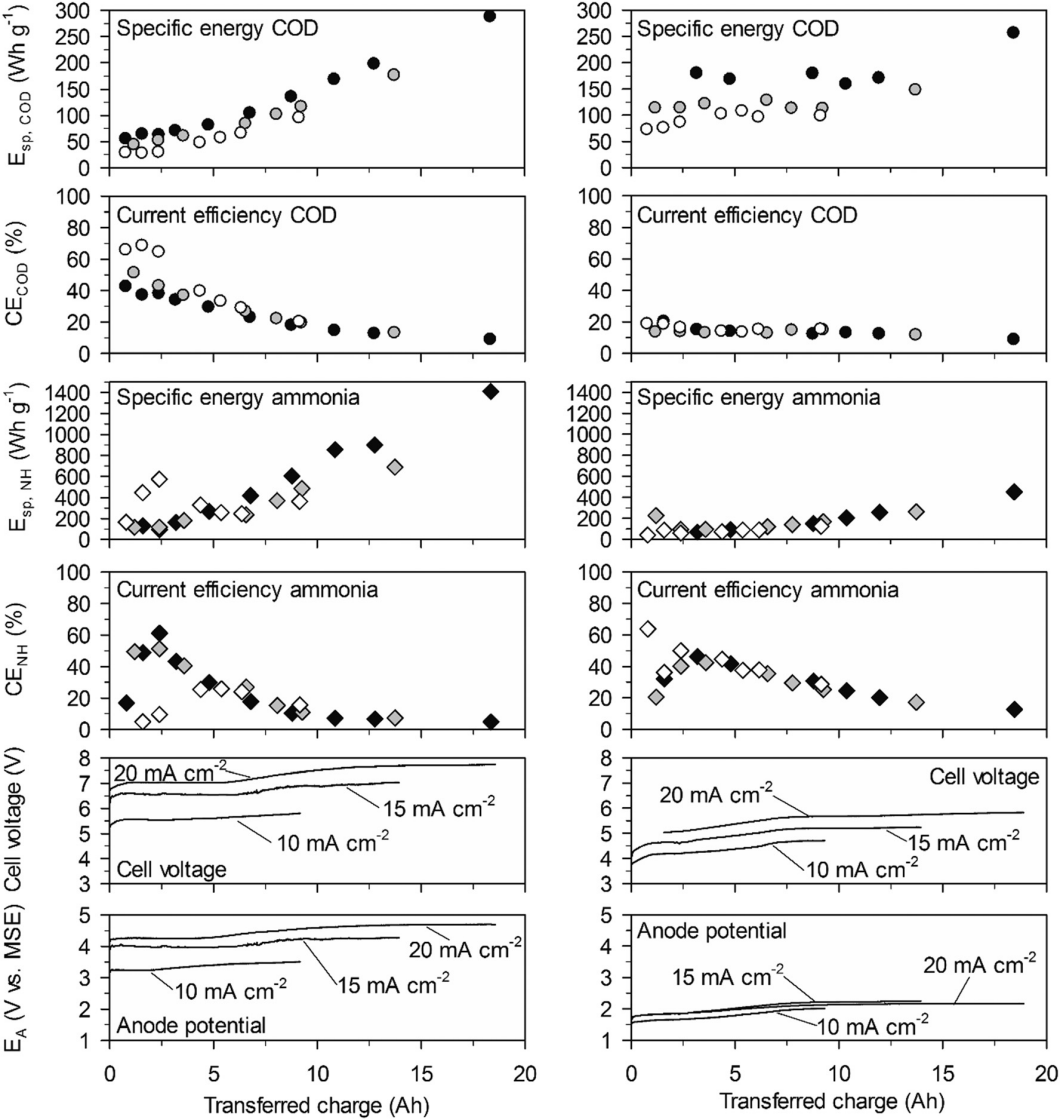
Cumulative specific energy consumption and current efficiency in low-concentration urine. Left: With the BDD anode. Right: With the TDIROF anode. Full symbols: 20 mA cm^−2^, grey symbols: 15 mA cm^−2^ and empty symbols 10 mA cm^−2^.

On BDD, the cumulative specific energy consumption for COD removal, *E*_sp,COD_, increased with the transferred charge (Fig. 6). At 20 mA cm^−2^, *E*_sp,COD_ increased from an initial value of 55 W h gCOD^−1^ to 104 W h gCOD^−1^, when 94% of the COD was removed and 6.8 A h were transferred. Within the same experimental period, the initial CE_COD_ dropped from 42% to 23%, while the cell voltage hardly changed. Thus, the twofold increase of *E*_sp,COD_ mainly resulted from a twofold reduction of CE_COD_. Furthermore, the *E*_sp,COD_ was higher than in highconcentration urine (Fig. 3) due to lower values of CE_COD_.

On TDIROF at 20 mA cm^−2^, the *E*_sp,COD_ was stable at a high level (∼170 W h gCOD−1) up to an elimination of 80% of the COD after 12 A h were transferred. This was a consequence of the near constant and low CECOD (∼15%, Fig. 6). These results confirmed the finding from the experiments in high-concentration urine that COD was degraded more efficiently on BDD due to the higher CECOD

The *E*_sp,NH_ was clearly smaller on TDIROF (Fig. 6). The *E*_sp,NH_ was 90 W h gN^−1^ on TDIROF at a current density of 20 mA cm^−2^ when the linear removal came to an end (5 A h). On the BDD anode, *E*_sp,NH_ was 270 W h gN^−1^ after the same transferred charge. The better *E*_sp,NH_ on TDIROF resulted from lower cell voltages and from the higher CE_NH_ which confirmed this finding from the experiments in high-concentration urine.

## 4 Discussion

### 4.1 Influence of the urine composition

The COD could be eliminated to low levels in all experiments as COD removal was faster with AC but could proceed also without AC. These AC independent processes were the oxidation of organic matter with hydroxyl radicals on BDD and the direct oxidation at the anode surface on TDIROF. Nitrogen, however, could not be eliminated completely and the final composition depended strongly on the initial composition of the urine. When the COD to chloride and, above all, the ammonia to chloride ratios were too high as in low-concentration urine (Table 1) ammonia was only partly oxidized.

In such cases, the residual nitrogen species can be explained with a previously established reaction system.^26^

$$CE_{N_{2}}\left(t\right)=\frac{\left(\Delta m_{NH_{tot}}\left(t\right)-\Delta m_{NO_{2}{}^{-}}\left(t\right)\cdot u_{e^{-},N_{2}}\cdot F\right)}{M_{N}\cdot Q\left(t\right)}$$ (11)

$$CE_{COD}(t)=\frac{\Delta m_{COD}\left(t\right)\cdot F}{TOD_{e^{-}}\cdot Q\left(t\right)}$$ (12)

$$2NO_{2}{}^{-}+4H_{2}O+6e^{-}\to N_{2}+8OH^{-}$$ (13)

$$NO_{2}{}^{-}+HClO\to NO_{3}{}^{-}+Cl^{-}+H^{+}$$ (14)

$$E_{sp,x}=\frac{\bar{U\cdot I}\cdot t}{\Delta m_{x}\cdot 3600}$$ (15)

$$Q\left(t\right)=\int_{0}^{t}I\left(t\right)\cdot dt$$ (16)

In addition to the oxidation of ammonia to N_2_ (eqn (11)) and nitrate (eqn (12)), the produced AC oxidized nitrite to nitrate (eqn (13)). Simultaneously, nitrate was electrochemically reduced at the cathode (eqn (14)–(16)).^27^ In presence of AC in the bulk, nitrite was immediately re-oxidized to nitrate resulting in nitrate accumulation as long as chloride was present. The AC concentration in the bulk got low when chloride oxidation ceased resulting in nitrite appearing in the bulk due to the still strong nitrate reduction at high nitrate concentrations. Simultaneously, nitrite was reduced more strongly to ammonia (eqn (15)) and N_2_ (eqn (16)), accelerated by higher nitrite concentrations in the cathodic diffusion layer. After a while, an equilibrium was established between nitrite oxidation to nitrate at the anode (eqn (14)) and nitrate reduction to nitrite at the cathode (eqn (14)) leading to a quasi-steady state. However, some nitrite was still reduced according to eqn (15) and very little according to eqn (16). In accordance with these processes, the pH value dropped during dominating ammonia oxidation to N_2_ and nitrate (eqn (11) and (12)) and increased when nitrite reduction to ammonia or N_2_ dominated (eqn (15) and (16)).

Complete ammonia removal was achieved with BDD at 20 mA cm^−2^ in high-concentration urine. However, complete ammonia removal should also be feasible on TDIROF in high-concentration urine. In fact, chloride would have been the last substance being used up based on an extrapolation of the removal rates for COD, ammonia and chloride (Fig. 2). The nitrogen remaining in the urine should than be mainly in the form of nitrate (between 7 and 50% may be expected).

### 4.2 Removal of COD and ammonia

#### 4.2.1 BDD

Very high specific COD removal rates were achieved on the BDD anode. They were two orders of magnitude higher than in biofilm systems (4 gBOD m^−2^ d^−1^).^28^ COD removal on BDD was also quite efficient at high COD concentrations, which can be seen from the high current efficiencies and the comparatively low energy demand in the beginning of the experiments (Fig. 3 and 6). The high current efficiencies resulted from a complex interplay of the oxidation *via* hydroxyl radicals and AC. Boudreau *et al*. proposed that a model organic substrate (acetaminophen) was first oxidized by AC and subsequently mineralized by an oxidation with hydroxyl radicals.^29^ Similar crossovers from the oxidation by hydroxyl radicals to the oxidation by AC likely happened in urine. In fact, it was evident from the results presented in an earlier work that especially short chain organic molecules were oxidized by indirect oxidation with AC because mainly short chain organic chlorination byproducts were formed.^19^ However, the much higher COD removal rates compared to the TDIROF anode must have resulted from the additional oxidation process *via* hydroxyl radicals as observed in other studies^25,30^ since chloride oxidation rates were mostly lower on BDD (Table 2).

Ammonia was only removed in the presence of chloride,which was clearly demonstrated in the experiments in low-concentration urine (Fig. 4 and 5). It can be concluded that indirect ammonia oxidation *via* AC was the responsible process. Neither direct oxidation at the electrode surface nor the oxidation by hydroxyl radicals happened to a great extent. The inhibition of direct ammonia oxidation might be explained with a pH drop in the Nernstian diffusion layer as was found for the TDIROF anode.^23^ This mechanism is reasonable for BDD as well since Kapałka *et al*. reported a high pH sensitivity for direct ammonia oxidation on BDD.^31^ Nevertheless, ammonia was completely removed with BDD although only from high-concentration urine with a low ammonia to chloride ratio (0.74 mgN mgCl^−1^, Table 1). In low-concentration urine, the ammonia to chloride ratio was higher (1.5 mgN mgCl^−1^, Table 1) such that not sufficient AC could be produced to remove all ammonia.

In high concentration urine at 20 mA cm^−2^, two phases with near constant ammonia removal rates could be distinguished. This could be due to the following mechanism. In the first phase, when COD concentrations were high, AC was mainly reacting with organic substances as explained above. Together with the lower AC production rates, this resulted in low indirect ammonia oxidation rates (Table 2). In the second phase, ammonia oxidation increased because more AC was available to react with ammonia as the COD concentrations were already at much lower levels.

#### 4.2.2 TDIROF

The removal of COD was clearly less efficient on TDIROF than on BDD. The oxidation *via* AC was the dominant process while chloride was available but direct oxidation at the electrode surface happened as well. In many other studies the presence of chloride was also found to have a strong impact on COD removal.^22,32^ Furthermore, the formation of chlorination byproducts demonstrated the reaction of AC with organic substances also in our own experiments.^19^

As on BDD, AC was inevitable for substantial oxidation of ammonia on the TDIROF anode (Fig. 5). Interestingly, carbonate oxidation did not outcompete chloride oxidation in real urine in contrast to the findings in synthetic urine by Amstutz *et al*.^21^ The reason for this could be the lower pH values and the lower total carbonate content in real stored urine (Table 1) leading to less CO_3_^2−^ which is the reactant for carbonate oxidation. Direct ammonia oxidation at the anode surface was probably largely inhibited by a pH drop in the Nernstian diffusion layer and could not contribute to ammonia removal.^23^

Nevertheless, the ammonia removal rates were clearly higher on TDIROF (Table 2) than on BDD and up to two orders of magnitude higher than in biofilm systems (2.5 gN m^−2^ d^−1^).^33^ This suggested a better availability of AC for the oxidation of ammonia on the TDIROF anode. The reason for this was that AC was not used up in reactions with short chain organic substances as on BDD. On TDIROF, the reactions of AC with longer chain organic substances were slower^19^ and resulted in more available AC for ammonia oxidation also at high COD concentrations. However, also with the TDIROF anode the ammonia to chloride ratio was too high in low-concentration urine (1.5 mgN mgCl^−1^, Table 1) to achieve complete ammonia removal.

### 4.3 Specific energy demand

#### 4.3.1 COD removal

The specific energy demand for COD removal (*E*_sp,COD_) depended on the cell voltage but mainly on the current efficiency (CE_COD_) in function of the COD concentration. The *E*_sp,COD_ value we found on BDD in low-concentration urine for a COD elimination of 94% (104 W h gCOD^−1^) was two times higher than what we found in high-concentration urine for an elimination of 90% (55 W h gCOD^−1^). The cause of this were much higher CE_COD_ values in high concentration urine, which points out once more the preferential COD elimination on BDD at high concentrations. An *E*_sp,COD_ value of 129 W h gCOD^−1^ was reported recently in synthetic urine with an initial COD concentration of 825 mgCOD L^−1^ (94% elimination).^34^ Compared to our findings, this result shows that the diverse organic substances in real urine are degraded as efficient as model COD compounds in synthetic urine.

On TDIROF, *E*_sp,COD_ was generally higher than on BDD but the dependence on the COD concentration was identical. COD removal on TDIROF required between 1.7 and 3 times more energy than COD removal on BDD even though the cell voltages were lower. The cause was the much lower CE_COD_ on TDIROF. This contradicts findings in synthetic urine by Dbira *et al*. who found a lower *E*_sp,COD_ (105 W h gCOD^−1^, 95% elimination) with an IrO_2_–RuO_2_ anode which might be caused by a different CE_COD_ in their experiments.^11^

Compared to other COD reduction technologies the energy demand of electrolysis was very high. In the partial nitrification of urine, the elimination of COD was observed as a side effect. Maurer *et al*. estimated an energy demand of 54 MJ m^−3^ for the removal of 82% COD, when the initial COD concentration was 10 000 mgCOD L^−1^.^3^ This corresponds to an *E*_sp,COD_ value of 1.83 W h gCOD^−1^. Other technologies cannot completely eliminate but reduce COD in the main urine stream. Electrodialysis could reduce COD by 90% requiring 30 kW h m^−3^ of energy.^3^ The resulting *E*_sp,COD_ value is 3.00 W h gCOD^−1^. Also evaporation has a lower energy demand to separate COD. A 99% removal of COD from the distillate required 400 MJ m^−3^ which corresponds to an *E*_sp,COD_ value of 11.1 W h gCOD^−1^.^3^

#### 4.3.2 Ammonia removal

On both anodes, *E*_sp,NH_ was not strongly influenced by the current density because similar current efficiencies were achieved with all current densities. The raw urine, however, had an impact on *E*_sp,NH_. In fact, the higher current efficiencies at high ammonia concentrations reduced *E*_sp,NH_. Comparing the two anodes, the removal of ammonia was clearly more energy efficient on TDIROF. The main reason for that was the low current efficiency for ammonia oxidation on BDD especially at high COD concentrations.

The energy demand for ammonia oxidation on TDIROF was in a similar range compared to other studies using electrolysis for ammonia removal in synthetic urine. Zheng *et al*. reported an energy demand for indirect ammonia oxidation of 73 W h gN^−1^ at an applied current density of 50 mA cm^−2^ with another type of DSA anode (RuO_2_–IrO_2_–TiO_2_/Ti).^20^ The same group estimated the energy demand for indirect ammonia oxidation with graphite anodes to 103 W h gN^−1^. Electrochemically, a lower *E*_sp,NH_ of 42 W h gN^−1^ was only achieved with direct ammonia oxidation on a graphite anode.^17^

Nitrogen could also be removed from source-separated urine with other technologies such as nitrification/denitrification with an organic electron donor or with the anammox process.^35^ However, urine contains too little organic substances for complete nitrification/denitrification.^36^ Only the anammox process was investigated in more detail but no experimental energy needs were reported.^37,38^ Maurer *et al*. estimated the required electrical energy for the anammox process with urine to 1.7 W h gN^−1^ which is considerably less than what was required for electrolysis.^3^

### 4.4 Applying electrolysis for urine treatment

Our experiments show that COD and ammonia can be removed from real stored urine with galvanostatic electrolysis. However, the simultaneous removal of ammonia and COD is only possible on TDIROF and in the presence of chloride. Nevertheless, the high removal rates indeed make electrolysis interesting for compact urine treatment reactors.

For a complete removal of organic substances and ammonia a serial combination of electrolysis cells could be interesting. In the first cell with a BDD anode, organic substances would be removed preferentially *via* the two oxidation pathways as described above. This would make more chloride available for the subsequent ammonia oxidation in the second cell that would preferably be equipped with a TDIROF anode to decrease the energy demand. In such a system it could also be possible to treat urine with unfavorable COD to chloride and ammonia to chloride ratios.

Despite these promising aspects of electrochemical COD and ammonia removal it must be noted that harmful byproducts were formed in oxidation processes with AC. In a recent publication, we have shown that the removed chloride was mainly oxidized to chlorate and perchlorate on both anodes depending on the duration of the treatment.^19^ After the complete elimination of COD and ammonia, it has to be expected that perchlorate is the dominant chlorine species. This poses a severe environmental risk.^39^ Furthermore, the study of Zöllig *et al*. showed that organic chlorination byproducts were formed which were mainly stripped into the gas phase.^19^

## 5 Conclusions

- On both anodes, ammonia can only be removed substantially by indirect oxidation with AC. However, COD removal also consumes AC, especially on TDIROF and to a smaller extent on BDD. Therefore, the ratios of COD to chloride and ammonia to chloride should be low to enable complete COD and ammonia removal. This means that stored urine with low ammonia concentrations, *e.g.,* due to ammonia volatilization, is more suitable for electrochemical nitrogen removal. Also, chloride dosage can enhance electrochemical ammonia removal.
- BDD is more efficient for the elimination of COD while TDIROF is better suited for the elimination of ammonia at high COD concentrations (above 1000 mgCOD L^−1^). The efficient COD elimination on BDD results from two oxidation pathways that aid one another. The oxidation *via* hydroxyl radicals produces smaller organic molecules which react efficiently with AC leaving little AC for ammonia oxidation. On TDIROF, the produced AC reacts with organics but also with ammonia.
- The difference in the reaction mechanisms on BDD and TDIROF anodes could be exploited by combining both electrodes in a serial reactor setup. The preferential oxidation of COD on BDD in a first cell should result in more available active chlorine for indirect ammonia oxidation in the subsequent cell with a TDIROF anode. In this way, complete COD and ammonia removal may be achieved even in urine with high ratios of COD to chloride and ammonia to chloride.
- Besides the formation of chlorination byproducts the high specific energy demand remains a main drawback of electrochemical COD and ammonia removal. It results from low current efficiencies and high cell voltages. The current efficiencies may be increased by decreasing the current density continuously with decreasing reactant concentrations. The cell voltage can be reduced by minimizing ohmic losses and by reducing the overpotentials at the anode and the cathode.
